# Group follow up proposal for elderly with hearing aids

**DOI:** 10.1016/S1808-8694(15)31171-X

**Published:** 2015-10-19

**Authors:** Eliara Pinto Vieira, Elisiane Crestani de Miranda, Lucila Leal Calais, Laura Maria Araújo de Carvalho, Maria Cecília Martinelli I ório, Alda Christina Lopes de Carvalho Borges

**Affiliations:** 1 Doctorate Student in Human Communication Disorders at UNIFESP/EPM, Speech Therapist at the Integrated Center for Attention, Research and Learning in Hearing at UNIFESP/EPM; 2 M.Sc, in Human Communication Disorders at Universidade Federal de São Paulo (São Paulo Federal University) UNIFESP/EPM, Speech Therapist at NIAPEA; 3 Doctorate Student in Human Communication Disorders at UNIFESP/EPM, Speech Therapist at NIAPEA; 4 M.Sc. in Human Communication Disorders at UNIFES/EPM, Speech Therapist at the Integrated Center for Attention, Research and Learning in Hearing at UNIFESP/EPM, at the Hearing Disabled Center (CDA) at São Paulo Hospital and the Teuto Brasileiro Hearing Center; 5 Professor at UNIFESP/EPM, Associate Professor at the Speech Therapy Department at UNIFESP/EPM; 6 PhD in Human Communication Disorders at UNIFESP/EPM, Associate Professor at the Speech Therapy Department at UNIFESP/EPM; São Paulo Federal University (UNIFESP) - Integrated Center for Attention, Research and Learning in Hearing at UNIFESP/EPM (NIAPEA)

**Keywords:** hearing, hearing aids, elderly, hearing disorder

## Abstract

**Summary:**

Implementing rehabilitation programs to hearing impaired adults is of great importance, mainly in the elderly population, and it is necessary to add them to the routine of outpatient care programs.

**Aim:**

to present a group care program for elderly patients who are fitted with hearing aids.

**Material and method:**

to carry out a pilot study of clinical and experimental type, with the participation of 40 elderly users of hearing aids donated by the government, distributed within six groups, with maximum of eight participants jointly with their respective companions. Program consisted of three meetings every fifteen days, where information and education on the proper use hearing aids was transmitted.

**Results:**

Most of the patients participated actively in the meetings spontaneously giving their opinion or answering questions when so requested. All elderly had been informed as to the importance of accepting their auditory deficiency and on the need to be motivated towards using hearing aids. Moreover, listening to depositions of other elderly users seemed to facilitate understanding of their own difficulties and stimulated them in the process of getting used to the sound amplification.

**Conclusion:**

Groups structure facilitated interaction among aged ones, helping them to clarify communication doubts and strategies and, consequently, it promoted their adaptation.

## INTRODUCTION

In the last century we have seen an increase in population longevity and demographic aging is a world trend.

According to data from the Brazilian Institute of Geography and Statistics (IBGE)^1^ in the year 2000 Brazil had 171,279,882 inhabitants, with 13,915,357 (8.1%) elderly people. The estimated general population for 2020 is 219,077,729, with 28,321,801 (12.9%) elderly people. The estimated number of elderly people for 2050 is equivalent to 24.7% of the total population.

With the development of medical technology, life expectancy increased and as a consequence there are much more senile illnesses and problems associated with this group of people^2^.

Aging is defined as a progressive and degenerative process characterized by less functional efficiency with the weakening of defense mechanisms. It is a universal and intrinsic process, that is to say, it is not determined by environmental factors despite their influence and it is different from diseases that many times are reversible and not seen in all old people in the same way^3^.

Among the several problems which affect the quality of life of the elderly, hearing problems are among the most common complications as it has been showed in several epidemiological studies.^4^, ^5^, ^6^, ^7^, ^8^

The term presbycusis refers to hearing loss as a consequence of the aging process^9^. This hearing loss is characterized by the result of different types of physiological degenerations associated with the effects of noise exposure, health problems and its treatment and genetic susceptibility^10^.

According to the literature^11^, ^12^, ^13^, ^14^, ^15^, hearing loss in the elderly is usually related to the deterioration of other sensory systems. Together with the hearing loss we can have the combined effect of multiple deficiencies, such as reduced cognitive performance, dementia, lack of manual skills and visual acuity.

Some authors^16^,^14^,^17^ report that hearing function deterioration on the aging process affects both communication and social and emotional domains, since it means a significant reduction in interaction and personal contacts.

Thus, social relationship of elderly people is affected by the disabilities of the aging process because they feel less valued and with less self-esteem. This gets worse with their difficulty to communicate.^18^

The old person who complains of hearing sensitivity must be sent for a complete audiological evaluation. However, it is necessary to state that audiometric measurements are not enough to describe the patient's reaction to hearing loss and the determination of communicative skills in their daily life and psycho-social function^19^. Thus, self-evaluation questionnaires are useful tools to quantify the emotional and social/situational consequences perceived due to hearing loss^20^,^7^.

When presbycusis and its consequences for the life of the elderly are verified, we notice the importance of hearing rehabilitation work in order to minimize the effects of hearing deficiency in the geriatric population^21^.

Getting used to a hearing device is hard at any age, especially for elder people. This is because elder people usually require more time to comprehend all the stages on the selection and adaptation process of amplification, and as a consequence, information should be presented gradually.

Education must be stressed so the user understands the way the hearing device works together with its components and the auricular mold. They must be trained in how to place it inside the ear and how to remove it, how to change batteries and to handle the control system^22^.

Thus, it is extremely important to implementat rehabilitation programs for hearing disabled adults and it is necessary to include these programs into the daily tasks of speech therapy outpatient clinics. These programs corroborate the elderly national policy which aims to guarantee the elderly social rights, preparing conditions for promoting their independence, interaction and effective participation in society^23^.

In order to illustrate this situation, here are some data from a Public Service on the diagnosis of hearing deficiency, selection and adaptation of hearing devices by the SIA-SUS (Public Health System) (APACS).

In this Service, 266 patients received a hearing device during March and July 2005. 120 (45 %) of them were elderly people, 46 (38.3%) were men and 74 (61.7%) were women. Out of the total number of old people 18 (15%) received a monoaural fitting and 102 (85%) received binaural fitting, with 222 hearing devices donated.

According to what it has been said above, this piece of work aims to show a group service program for elderly people with hearing devices.

## METHOD

A research and outreach project was prepared with the proposal of this Service and the Research Ethics

Committee of a Further Education Institution approved it with protocol number 1107/05.

Following ethical and legal principles, elderly people signed a Free and Informed Consent to take part in the project which had information on this service, such as its goal, justification, benefits for the participants and additional information.

A pilot study was done to evaluate the feasibility of this program. The sample included 40 elderly people with 15 men and 25 women aged between 60 and 90 years old (average age 76.3 years old) and who had received a device on this Service through the Public Health Care

**Appendix A**

**1°Questionnaire for elderly people**

Name: _________________________________________ Date: ___ / ___ /___

Age: ___________________ Gender: ( ) female ( ) male

1) What is your general health like?
  ( ) Do you have any vision problems, do you wear glasses ( ) good general health ( ) diabetes, high blood pressure, _____________________
2) For how long do you use the hearing device each day?
  ( ) Until 4 hours a day ( ) between 4 and 8 hours a day ( ) more than 8 hours a day
3) Did you complain of ringing in your ear before you started using the hearing device?
  ( ) no ( ) yes
  If you answered yes, did your complaint improve with the use of the hearing device?
  ( ) no ( ) yes
4) Which difficulties did you face during the adaptation process to the hearing device?
  ( ) handling and cleaning the device ( ) keeping a conversation in a noisy place ( ) understanding what people say ( ) talking on the phone
  ( ) your own voice sounds different ( ) sounds are too intense
  ( ) sounds are too weak
5) Which are the benefits you had with the use of the hearing device?
  ( ) improved social and/or professional life
  ( ) improved dialogue with relatives and friends
  ( ) the chance of watching TV or listening to the radio in a pleasant intensity
  ( ) talking on the phone without great difficulties
  ( ) better relationship with relatives and friends
  ( ) improved self-esteem and quality of life
6) In which items you still have some doubts or would like to have further information:
  ( ) hearing loss at an old age
  ( ) strategies to facilitate communication
  ( ) care with the hearing device (cleaning/maintenance/batteries)
  ( ) others, which one: ________________________________________

**Appendix B**

**Questionnaire for assistants**

Name: _________________________________________ Date: ___ / ___ /___

Kinship: ________ Age: ________ Gender: ( ) female ( ) male

1) Do you believe the hearing device brought benefits to your _________ ?
  ( ) no Why? ( ) does not use the device correctly
  ( ) he/she says is not adapting himself/herself to the use of the device
  ( ) other reason: ______________________
  ( ) yes which one? ( ) improved social and/or professional life
  ( ) watches TV and listens to the radio in a pleasant intensity for everyone at home
  ( ) improved dialogue with relatives and friends
  ( ) better relationship with relatives
  ( ) improved self-esteem and quality of life
  ( ) started doing things he/she did not used to do before using the device (for example __________)
2) Your relative/friend complains about something related to the use of the hearing device? ( ) no ( ) yes
  If you answered yes, which are the complaints?
  ( ) handling and cleaning the device
  ( ) difficulty in talking on the phone
  ( ) keeping a conversation in a noisy place
  ( ) sounds are too weak
  ( ) understanding what people say
  ( ) sounds are too intense

**Appendix C**

**How to take good care of your hearing device**

Your hearing device is a delicate instrument and it should be handled with care in order to work efficiently.

Pay attention:

Dust, heat, humidity and wax can damage your device!

Avoid dropping your device, this could be fatal!

Rinse it with a soft and dry cloth and do not forget: never use water to clean it.

Store the device inside your case when you are not using it and remove the battery. Keep it in a safe place.

Your device does not work without batteries. They provide the energy for the device to work. In order to make the best use of your batteries please follow the instructions below:

Remove the stamp of the battery and wait a moment to activate the charge.

Only remove the stamp of the battery when you are really going to use it;

Watch the sign “+” and “-” on the battery to insert it correctly;

Do always have batteries handy for the hearing devices.

In case your device is retro auricular (the one that is placed behind the ear), you should handle the mold as follows:

Remove and gently place the mold on the ear;

Always keep the mold clean!!! Wash it with water and soap frequently but before doing so detach it from the device, because the device cannot get wet.

After cleaning it, rinse the mold and the plastic tube well, always using a syringe to remove the remaining water.

Do not use any chemical product to clean the mold, only water and soap!

Change the mold and the plastic tube every time they are too dry, torn or yellowish.

**Appendix D**

**Helpful hints for the elderly user of hearing devices**

Some strategies that facilitate your communication:

Always look the person you are talking to in the face because you can use the visual hints.

Make the place where you are talking brighter and lower the noise. It will be difficult to talk if the TV or the radio is on very loud.

Let other people know about your difficulty to hear and try not to hide your hearing disability because people can try to look for other ways of communicating better with you.

Be always alert!!! Pay attention to the speaker, watch his/her face and facial and body expressions.

Try not to interrupt very often the person who is talking, pay attention, give him/her a sign to speak slower and/or increase a little bit the intensity of his/her speech.

Be patient and keep your sense of humor!!! Try not to quarrel with your family over communication difficulties. A quite family environment favors dialogue!!!

ALWAYS remember the importance of using your hearing device since it can benefit you a great deal. However, hearing devices have their limitations.

**Helpful hints for the family**

Some ways to behave with a person using a hearing device:

First call his/her attention, be sure you drew the attention of the person with hearing difficulties before you start talking.

Speak face to face to the person with hearing difficulties, giving him/her visual hints.

Make the place where you are having a conversation brighter because your face will be lit. But remember not to stand facing a light source (such as a window for example), because it will be difficult for him/her to see your face;

Do not place obstacles in front of your face: always talk with your mouth empty, because you will be helping him/her with visual hints of your lips.

Speak clearly and reduce the speed.

Do not shout!!! Just talk a little bit above your usual intensity.

Use facial expressions and gestures; however, do not exaggerate in your speech articulation.

Reduce the noise of the room: avoid having a conversation if the TV or the radio is on.

Re-phrase or instead of repeating, if the old person does not seem to have understood what you said, re-phrase the sentence very calmly!!!

Ask how you can help: ask the old person for suggestions on ways to improve communication.

Be patient and keep your sense of humor!!!

A quiet family environment favors dialogue!!!

ALWAYS encourage him/her to use the hearing device because it benefits the user and your family a great deal.

**Appendix E**

**2°Questionnaire for elderly people**

Name: _________________________________________ Date: ___ / ___ /___

1) If your device “beeps”, when in your ear, what should you do?
  ( ) check the placement of the device or mold (if it is a retro auricular device)
  ( ) check the volume (if it has volume control)
  ( ) clean the device
  ( ) change the battery
  ( ) wait until it stops beeping
  ( ) if none of the above helps, ask a speech therapist for help
2) In case your device is “weak”, what can you do?
  ( ) change the battery
  ( ) check the volume button (if it has this control)
  ( ) gently knock your device
  ( ) wait until the device starts working correctly again
  ( ) if none of this helps, ask a speech therapist for help
3) In case your device is not working, what should you do?
  ( ) check if the device is on
  ( ) check if the batteries are new
  ( ) check if the box is completely closed
  ( ) wait until the device starts working correctly again
  ( ) if none of the above helps, ask a speech therapist for help
4) Do you still have any doubts on your hearing device?
  ( ) no ( ) yes Which one? _____________________________
  5) Are you happy with your hearing device?
  ( ) yes ( ) no Why? ____________________________
6) Are you using the communication strategies offered by the interns at the group meetings?
  ( ) no Why? ________________________________________________
  ( ) yes
  If you answered yes, did it benefit your communication?
  ( ) no ( ) yes
7) Do you believe group meetings were useful?
  ( ) no Why? _______________________________________________
  ( ) yes Why? _______________________________________________
  System (SUS).

Inclusion criteria were as follows:

• Minimum age 60 years old (a criteria established by World Health Organization for developing countries);
• Time availability to attend the meetings after the donation of hearing devices;
• To live near the Service (Southern area of the city of São Paulo-SP);
• Without any moving disabilities.

The elderly people using hearing devices were divided into six groups with a maximum number of eight with their own relatives and/or assistants. The distribution of the elderly people into small groups aimed to offer them a better attention and to facilitate interaction among participants.

The program included three fortnightly meetings coordinated by speech therapists at the Service who use audio-visual resources and group techniques to share necessary information and orientations with the elderly. Each meeting had different goals.

The first meeting was held one week after the hearing devices were donated and had the following goals:

a) Introducing the participants of the group;
b) Explaining how group service works, clarifying the importance of their presence at all meetings and the need of having a relative or a close person attending the meetings together with the elderly person.
c) Explaining the following questions: What is hearing loss? Which are the degrees of hearing loss? What are hearing devices for? What is the reason for binaural or monoaural fitting? In order to make explanations easier, professionals used slides with the average acoustic values of frequency and intensity of Brazilian Portuguese speech sounds, displayed in the graphic registry of the audiogram24. Thus, it was possible to show each person what his/her hearing loss configuration was like, proving which sounds were audible without the hearing device and which he/she started to hear after using the device.
d) Strengthening guidance on how the hearing device works and how to clean it and maintain it.
e) Two questionnaires were used, one to be answered by the elderly person and the other one for a relative and/or assistant, aiming to compare the perceptions of both on the difficulties and the benefits regarding the use of the hearing device (Appendix A and Appendix B).

Questionnaires were specifically prepared for this study and they were given to the group by the responsible speech therapists, however each old person had the opportunity to voice his/her opinion. During the questionnaire old people did not have access to alternative answers.

Questionnaires had questions with multiple choice questions and others had open questions. For those multiple questions, the speech therapist selected, among the alternative answers, the ones corresponding to those mentioned by the elderly people. On the other hand, for the open questions, the speech therapist transcribed the answer given by the elderly person.

At the end of this first meeting, participants received an information brochure specially prepared for this program with clearly written orientations (Appendix C).

The second meeting was held 21 days after the hearing devices were given and it had the following goals:

a) Promoting the exchange of opinions on the impact of hearing disability and hearing devices fitting at an old age with their statements on how they experienced the use of devices, their main difficulties and benefits until this moment, among other issues.
b) Offering facilitating communication strategies for elderly people, besides giving them an information brochure at the end of the meeting with communication guidelines for people with hearing disabilities^25^ (Appendix D).

The third meeting took place 35 days after the hearing devices were given and it had the following goals:

a) Promoting the reading and further discussion of a text on social isolation experienced by old people with communication problems as a consequence of a hearing disability;
b) Clarifying the possible doubts they may still have on adaptation to the hearing device;
c) A new questionnaire also done specially for this program aiming to evaluate the understanding of the guidelines offered during the group meetings. (Appendix E).

## RESULTS AND DISCUSSION

Most elderly people actively participated in the meetings, expressing their opinions in a spontaneous way or answering the questions when they were asked. All of them were guided on the importance of accepting hearing loss and on the need of being motivated to use the hearing devices^22^. Besides, listening to other people stories seemed to facilitate the understanding of their own difficulties and it encouraged them during the adaptation process to amplification.

Similarly to literature^26^, we observed that the support group is important for developing skills and for building up trust to interact with other people, making the whole inter-personal communicative process much easier.

Furthermore, speech therapists can introduce strategies to facilitate communication, to strengthen guidance on how to take care, handle and clean hearing devices and/or auricular molds, and also they can provide patients with information on hearing.

Regarding communication strategies, some authors^21^,^27^ state that the use of these devices facilitates speech understanding and having a dialogue which keeps the communication process active.

In regard to questionnaires, they have been used to evaluate the benefit, use and satisfaction of people with hearing devices, as well as in following up the adaptation process^20^,^7^. Thus, it was possible to evaluate the patient's communicative performance after receiving the device and to get to know his/her opinion on the main difficulties and benefits experienced in daily situations, as it has been showed in the literature.^28^. It was also possible to considerate the usefulness of the program.

In the last meeting we discussed social isolation due to communication difficulties. One of the participants, a 78 year old lady said that “.. I used to watch TV very loud and no one in my family would stay in the room… I stopped attending an elderly group because I didn't hear what the coordinator said. I couldn't understand or talk to the doctors on my visits…”. This lady's report clearly shows the social difficulties these elderly people experience, a fact already commented by several authors^18^,^16^,^14^,^17^.

## CONCLUSION

Group work was useful for the interaction among the elderly people and it made possible to clarify doubts and communication strategies, favoring the adaptation process.

It is believed that more studies on the effectiveness of this type of service proposal are needed, since there are an increasing number of registered services for the donation of hearing devices by the Public Health System (SUS) and therefore, geriatric population increasingly demands this service.
